# Squamous Cell Carcinoma of the Rectum 21 Years after Radiotherapy for Cervical Carcinoma

**DOI:** 10.4103/1319-3767.54745

**Published:** 2009-07

**Authors:** Kevin K. Leung, Joseph Heitzman, Anand Madan

**Affiliations:** Department of Gastroenterology, The University of Texas Health Science Center, Houston, TX 77030, USA; 1 Department of Pathology and Laboratory Medicine, The University of Texas Health Science Center, Houston, TX 77030, USA

**Keywords:** Human papilloma virus, radiotherapy, rectum, squamous cell carcinoma, surgery

## Abstract

Squamous cell carcinoma (SCC) of the rectum is an extremely rare malignancy, accounting for 0.1-0.2% of rectal malignancies. It is associated with ulcerative colitis, prior radiation, schistosomiasis, ovarian cancer, endometrial cancer, human papilloma virus, colocutaneous fistulas and colonic duplication. Prior reported cases of SCC of the rectum have involved treatment with brachytherapy and external beam radiation. This case is particularly interesting because of the remote exposure of radiation (21 years previously) and the subsequent development of SCC of the rectum. Although extremely rare, SCC of the rectum can occur decades after radiation exposure.

Squamous cell carcinoma (SCC) of the rectum is extremely rare, accounting for approximately 0.1-0.2% of all rectal malignancies.[1] It was first described in 1919 by Schmidtmann.[2] Based on previous case reports, other conditions have been associated with this rare malignancy, such as colocutaneous fistula,[3] prior radiation,[4,5] ulcerative colitis,[6–8] schistosomiasis,[9] ovarian cancer,[4] ovarian teratoma,[10] endometrial carcinoma,[11] human papilloma virus (HPV)[12] and colonic duplication.[13,14] Previously reported cases of SCC with a history of radiotherapy have involved brachytherapy for the treatment of prostate cancer[5] and for the treatment of ovarian adenocarcinoma.[4] We present a very rare case of SCC of the rectum that, to our knowledge, is the first reported case in which this tumor was diagnosed 21 years after radiotherapy for cervical carcinoma.

## CASE REPORT

**Figure 1:**
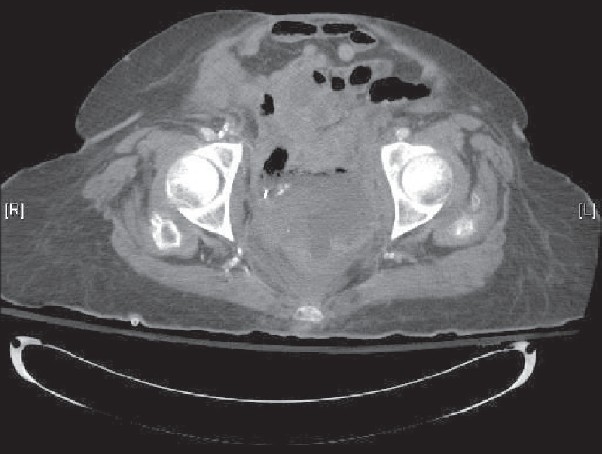
Abdominal CT scan showing bladder infiltration from the squamous cell carcinoma of the rectum

**Figure 2:**
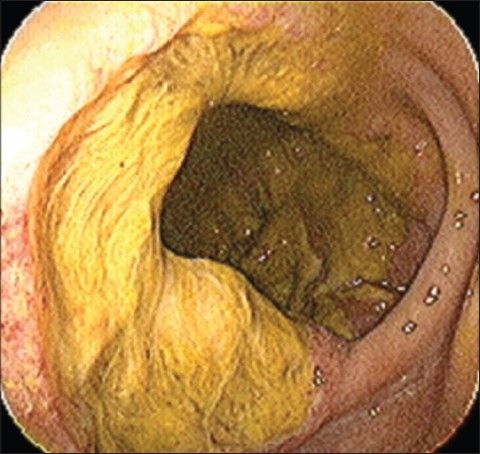
Endoscopic view of rectal mass

**Figure 3:**
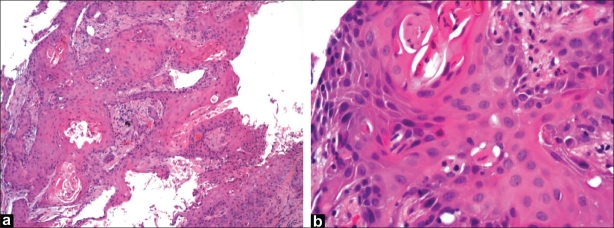
(a) (H and E, ×10) and E section of the rectal biopsy shows well differentiated squamous cell carcinoma with areas of keratinization that has become invasive. The typical columnar epithelium with goblet cells has been replaced with the neoplastic process into the submucosa; (b) (H and E, ×40) and E section of the rectal biopsy shows higher magnification of the neoplastic cells with large round nuclei with prominent nucleoli, along with the formation of keratin “pearls” seen in well differentiated squamous cell carcinoma

A 63-year-old Hispanic woman was admitted to the hospital for urosepsis. She had a past medical history of cervical cancer treated with radical hysterectomy and radiotherapy in 1986. There was no evidence of residual cervical cancer or a history of inflammatory bowel disease. Because of her history of malignancy, complaints of pneumaturia of 2 months duration, polymicrobial blood and urine culture results and 30 lb weight loss over 1 year, an abdominal computed tomography (CT) was performed. It revealed a 9 cm pelvic mass with vesicocolonic fistulization, multiple hepatic lesions consistent with metastatic disease and local bony invasion of the pelvis [Figure 1]. A colonoscopy was performed, which revealed a large circumferential ulcerated and friable mass with the distal aspect located 3 cm proximal to the dentate line [Figure 2]. There was no involvement of the anorectal junction. Pathology showed a well-differentiated invasive SCC [Figure 3a and b]. Immunohistochemistry was negative for HPV and cytokeratin 7. Chest CT was negative and no other source for malignancy was identified. Because of her metastatic disease and refusal of palliative chemotherapy, a diverting colostomy was successfully performed and she was subsequently transferred to home hospice.

## DISCUSSION

In 1919, Schmidtmann reported the first case of SCC of the colon.[2] Given the rarity of this malignancy, a majority of the published cases are case reports. However, a few case series from large institutions involving a small number of patients have been published. Frizelle *et al.*[15] reviewed the Mayo Clinic tissue registry spanning 85 years and identified only 11 cases of SCC.

In 1979, Williams *et al.*[16] proposed three criteria that should be met before establishing a diagnosis of SCC: (1) metastasis from other sites to the colon must be excluded, (2) squamous-lined fistula tract must not involve the affected bowel because it may be a source of SCC and (3) SCC of the anus with proximal extension must be excluded.

The etiology of SCC is unclear. Several theories have been proposed to explain the development of SCC, including: (1) proliferation of uncommitted stem cells into squamous cells that subsequently undergo malignant transformation, (2) squamous metaplasia resulting from chronic inflammation, (3) squamous differentiation of adenomas and adenocarcinomas and (4) pleuripotent stem cells capable of multidirectional differentiation.[17]

Traditionally, surgical resection has been the most appropriate and effective treatment.[17] Because of the rarity of this malignancy, the role of chemoradiation has not been clearly defined.[10,18,19] The prognosis is similar to rectal adenocarcinoma for Stage I and II node-negative disease; however, nodal involvement is associated with a worse prognosis than adenocarcinoma of a similar stage.[7] Adjuvant chemotherapy or chemoradiation, although unproven, should be considered in conjunction with surgical resection.

There have been limited reports of SCC of the colon arising after radiotherapy.[4,5] In this patient, she was previously treated with radiation 21 years ago for cervical carcinoma. Late recurrence of cervical cancer has been reported 26 years after initial therapy.[20] However, our case unlikely represents a late recurrence. Immunohistochemical staining for HPV was negative, virtually excluding a recurrence of cervical carcinoma.[21,22] Furthermore, cytokeratin 7 immunohistochemical staining should be positive if the malignancy was of gynecological origin.[23] Replacement of the rectal lining with squamous epithelium has been reported following repeated exposure to radiation.[24]

One of the patient's initial symptoms was pneumoturia. Vesicovaginal fistulization is a well known complication from radiotherapy, occurring between 4% and 47.8%.[25–27] However, given the long time period between radiotherapy and presentation of pneumoturia, the fistula tract was probably caused by bladder invasion by the SCC of the rectum.

In conclusion, this case highlights a rare and interesting case of SCC of the rectum resulting from radiotherapy 21 years earlier. Although extremely rare, SCC of the rectum can occur decades after radiation exposure.
